# Assessing Motion Fidelity During Closed-Head Rotational Acceleration Traumatic Brain Injury in Pigs

**DOI:** 10.21203/rs.3.rs-10806947/v2

**Published:** 2026-09-01

**Authors:** Susan Shin, Kevin D Browne, Olivia Amador, Declan A Patton, Michael Rosalino, John A Wolf, Kristy B Arbogast, David F Meaney, D Kacy Cullen, Kathryn L Wofford

**Affiliations:** University of Pennsylvania; University of Pennsylvania; University of Pennsylvania; University of Pennsylvania; University of Pennsylvania; University of Pennsylvania; Children’s Hospital of Philadelphia; University of Pennsylvania; University of Pennsylvania; University of Pennsylvania

**Keywords:** traumatic brain injury, closed-head diffuse TBI, rotational acceleration, rotational velocity, injury kinematics, DeepLabCut

## Abstract

**Purpose:**

Traumatic brain injury (TBI) affects 69 million people annually and is most frequently caused by rapid rotational acceleration of the head. Modeling rotational acceleration injuries in pigs can employ biomechanical parameters scaled from humans, allowing researchers to answer basic and translational questions about the influence of injury kinematics on outcomes. However, precisely describing head kinematics during rotational acceleration injuries is imperative to understand the relationships between biomechanical parameters, pathology, and neurological outcomes.

**Methods:**

Using an established model of rapid rotational acceleration, we examined kinematic transfer efficiency by assessing how closely the motion of the subject’s head matched the motion of the rotational device. Pigs were submitted to head rotation in the sagittal plane at either a moderate or a high target velocity. Kinematics were recorded with transducers and high frame rate videography, which was quantified manually and with the deep learning neural network, DeepLabCut. The rotational device was programmed to generate peak angular velocities ranging from 85.8–108.1 rad/s.

**Results:**

The kinematic coupling of the peak angular velocities between the head and transducer-mounted injury device was 95.30 ± 3.41% during moderate velocity TBIs and 93.09 ± 8.08% during high velocity TBI. Angular velocity exhibited strong matching between the head and injury device while angular acceleration exhibited fair-to-strong matching, depending on the kinematic term. These angular velocity and acceleration levels exceeded clinical injury thresholds from the literature based on appropriate mass scaling.

**Conclusions:**

The high degree of kinematic matching between injury device and head in both moderate and high velocity TBIs suggests a high kinematic transfer efficiency within the target range of angular velocities. This validation study provide critical insights into the fidelity of large animal rotational acceleration injury by enhancing our understanding of force transmission and head kinematics, leading to more accurate scaling and representation of human TBI events.

## Introduction

Traumatic brain injury (TBI) is a discrete biomechanical event that damages brain tissue, potentially causing neurological consequences, long term disability, and death [1]. Head biomechanics during the TBI are directly linked to the extent and distribution of pathology within the brain [2–5]. The most common form of clinical brain injury is a closed-head diffuse TBI, caused by rapid rotational acceleration [6, 7]. A pig model of closed-head rotational acceleration TBI has been used in the field of neurotrauma to mimic the biomechanics, pathology, and physiology of the most common form of TBI in humans [2, 4, 8–15]. Given the mass and neuroanatomical features, this large animal model of TBI more closely replicates the mechanics of common clinical brain injuries relative to other injury models [3, 4, 16]. The closed-head rotational acceleration model can generate varying injury severities depending on the peak angular velocity of the rotational device [10, 17]. However, since kinematics, or descriptors of rotational motion, are typically collected from the rotational device, not the subject, it remains unclear what proportion of the loading is transmitted to the subject during this rotational injury. Indeed, recent research by Mayer *et al*. [18, 19] and Patton et al. [20] has shown that the movement kinematics of the rotational device overestimates the head’s movement kinematics in the coronal plane. However, this has yet to be investigated for rotational injuries in the sagittal plane. Thus, the goal of this study is to utilize high frame rate videography to quantify the coupling between the rotational device’s movement and the subject’s head movement, which we refer to as kinematic fidelity, across a range of injury velocities in the sagittal plane.

## Methods

All procedures were approved by the University of Pennsylvania’s Institutional Animal Care and Use Committee (IACUC) and adhered to the guidelines set forth in the Public Health Service Policy on Humane Care and Use of Laboratory Animals (PHS Policy, 2015).

### Animal enrollment

For this study, a total of 5 female Yucatan pigs (6 months old) were enrolled. Yucatan pigs were procured from Sinclair Bio Resources (Auxvasse, MO) and were quarantined for 3 days upon arrival to the vivarium with access to standard food and water ad libitum. Subjects were randomly assigned to one of two levels of angular velocity injury in the sagittal plane: target range 85–90 rad/s (n = 2) or target range 105–110 rad/s (n = 3). No sham animals were used as this study aimed to measure kinematic fidelity during an injury experience.

#### Closed-head diffuse brain injury

**Figure 1.**
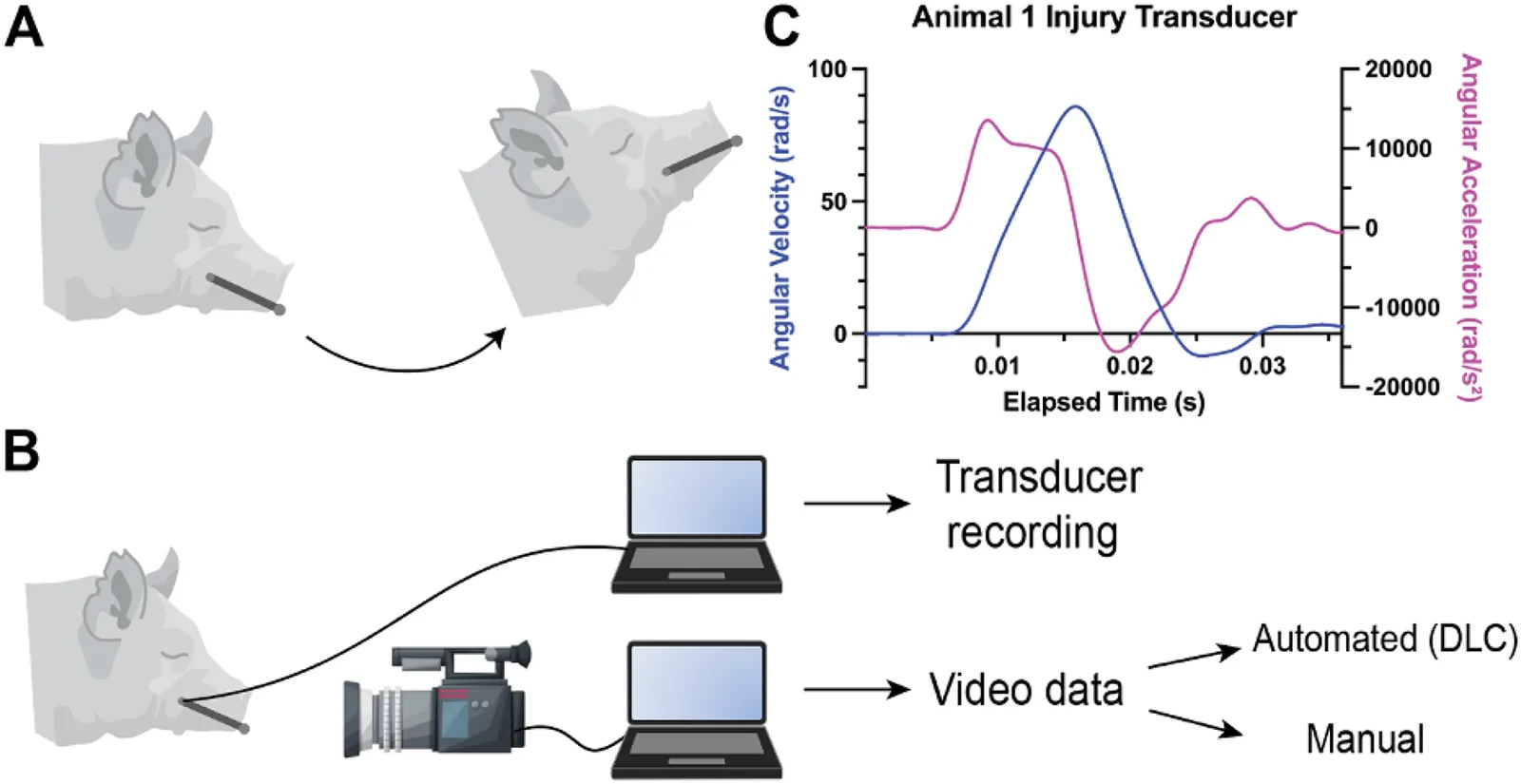
Closed head rotation injury is generated by rapidly rotating the head the sagittal direction (A). A schematic of data collection methodologies (B). An example angular velocity and acceleration curve where the blue curve shows the averaged angular velocity from two transducers in rad/s over the course of the injury and the magenta curve shows the averaged angular acceleration from two transducers in rad/s^2^ over the course of the injury (C).

Once cleared from quarantine, animals were fasted 12 hours prior to the procedure. Anesthesia was induced via intramuscular administration of midazolam (0.4–0.6 mg/kg) and ketamine (20–30 mg/kg). Animals were intubated and anesthesia was maintained via 2–5% isoflurane in 1.5–2.5 L/min oxygen. Anesthetic level, blood oxygen saturation, temperature, heart rate, and respiratory rate were monitored throughout the process and kept within normal limits through titration of anesthetic [21]. Brain injury was delivered using a pneumatic device that places the head to rapid angular acceleration (HYGE Inc., Kittanning, PA). Briefly, compressed nitrogen drives a vertical piston, which in turn is connected to custom-assembled linkages that convert the linear motion of the thrust column to the angular motion experienced by the head. For a more complete description of the HYGE device and its operation, see Cullen *et al*. (2016). Once the animal reached a surgical plane of anesthesia, the head was mounted onto a padded bite plate connected to the HYGE device and the snout was securely fastened to the bite plate with snout cables. For injury in the sagittal plane, the head was rapidly rotated 45° upwards starting from approximately 30° below the horizontal, which produces impulsive movement that can be scaled to humans (Fig. 1A) [5, 10]. While this method generates both rotational and linear acceleration, rotational acceleration is generally considered the primary biomechanical mechanism generating injurious forces and tissue deformation in the brain [10, 22, 23]. Upon completion of the injury, animals were recovered and returned to the home cage. Extended release buprenorphine was provided for analgesia.

#### Kinematic Recordings

**Figure 2.**
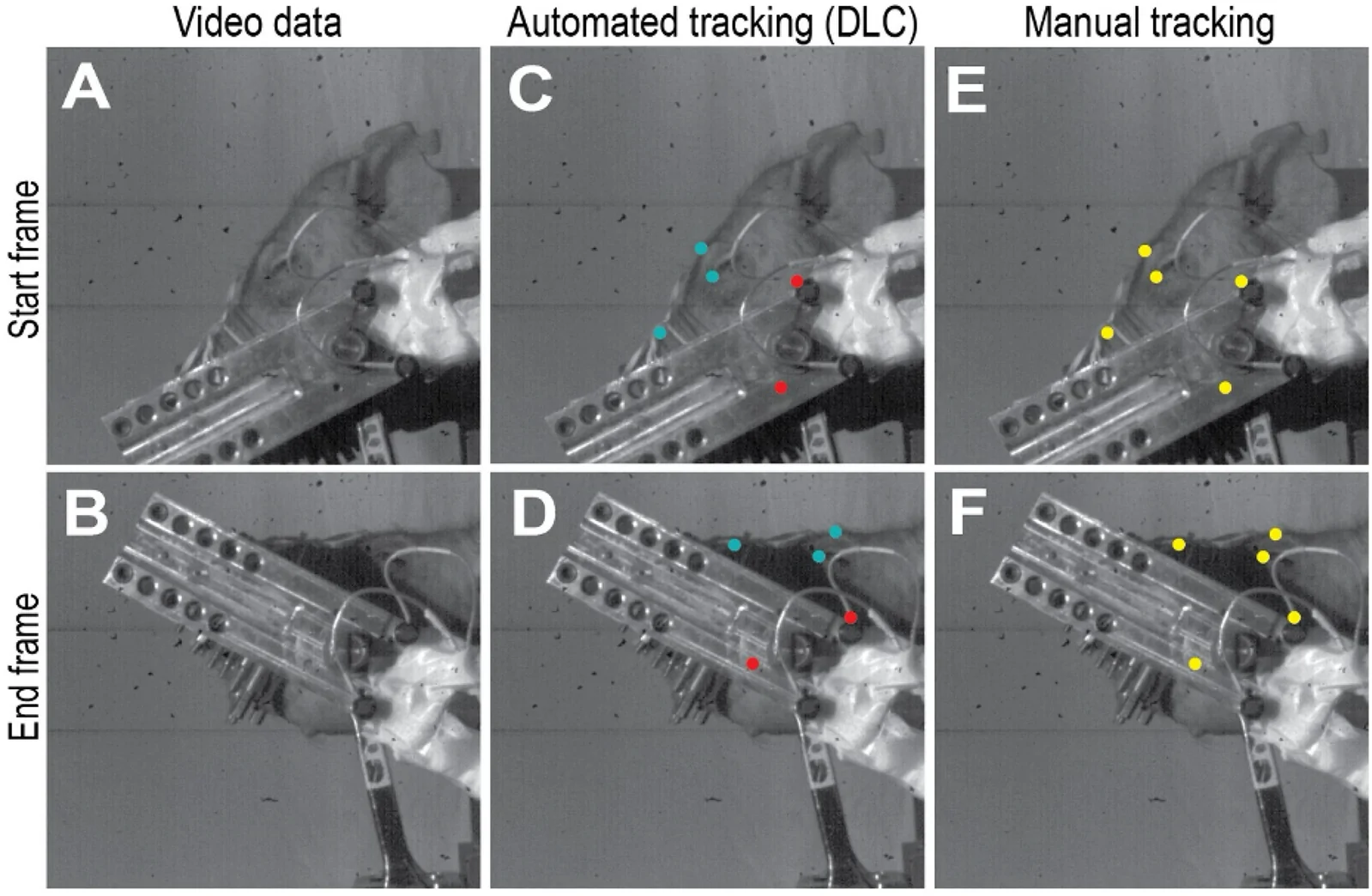
Example of videography data and head position in the start frame (A, C, E) and end frame (B, D, F). Example of automated DLC identification of three head anatomic landmarks (cyan) and two machine anatomical landmarks (red) in start frame (C) and end frame (D). Example of manual identification of head and machine anatomical landmarks in start frame (E) and end frame (F).

Injury kinematics were recorded using two independent methods: high frame rate videography and magneto-hydrodynamic sensors (Fig. 1B). High-speed videography was taken by a Phantom v4.2 Vision Research camera with AF Micro-Nikkor 60mm f/2.8D Lens. Independent optical evaluations of this lens report negligible geometric distortion (0.03%) [24]. The camera was positioned perpendicular to the rotational device to ensure that the motion exists primarily in the camera plane. The high-speed camera recorded data at a sample rate set to 500 fps with 0.94 ms of exposure time. The frames were recorded to the camera’s circular memory buffer (RAM) for the duration of the trigger signal, yielding a pre-set number of 958 frames. The camera was manually triggered, so the breakdown of pre- and post-trigger varies across trials. However, within all of these manually triggered videos, the entire duration of the rotational movement was captured. The Phantom Control Unit computer received the data from the camera to process the frames into TIFF files, which was later processed into a mp4 video. No files were compressed, altered, or distorted to influence the recorded motion in the video (Fig. 2). Care was taken to ensure the rotational device and the apical point of the pig’s head were visible throughout the range of rotational movements.

Separately, the transducer kinematics were recorded from two magneto-hydrodynamic sensors (Applied Technology Associates, Albuquerque, NM). Two transducers were bolted to the HYGE rotational device and connected via cables to a recording computer, as the historical data acquisition method which has been previously described [4, 7, 8, 10, 13, 15, 25, 26]. The voltage data from the transducers were recorded in real time and converted to angular velocity using the software LabVIEW at a sampling rate of 10 kHz (Fig. 1C). The two sensors were calibrated to 22.9 mV/rad/s and 22.1 mV/rad/s, respectively, to allow instantaneous recordings to be visualized in angular velocity (rad/s) immediately after the capturing. The average of two sensors was used to denote transducer velocity.

To mitigate signal noise, a fourth-order low-pass Butterworth filter with a cutoff frequency of 300 Hz was applied to the angular velocity data using Butterworth function in MATLAB R2024b (MathWorks – Natick, MA; Supp. Figure 1). Angular acceleration was calculated from the slope of the angular velocity curve. The cutoff frequency was informed by the Channel Frequency Class (CFC) 180 filter presented in the SAE J211 standards [27–30]. This type of filter has been used for head impact data in sports and pigs [20, 28, 29]. Similar thresholds have been used in sports [31, 32]. To confirm the desired signal was within the chosen cutoff frequency, the transducer data were converted into the frequency domain by calculating the fast Fourier transform (FFT), and a subsequent power spectral density analysis was performed. This analysis showed prominent peaks below the 300 Hz cutoff frequency, indicating that the desired signal’s power was localized within the passband and would be preserved in the low-pass filter.

#### Manual image analysis

The open-source software Fiji (version 2.14.0/1.54f) was used for manual image analysis. Five easily identifiable anatomical landmarks were selected based on visibility throughout the frames of injury. Three points on the animal’s head were placed on the snout, forehead, and corner of eye (Fig. 2E–F). These anatomical locations were visible in most video frames and minimal skin movement relative to the skull was qualitatively determined. Two points on the HYGE arm were placed on the screw head and top transducer’s cable (Fig. 2E–F). Visual accuracy was observed frame by frame to evaluate validity of point selection and adjustments were made if necessary. Points were subjectively assessed for edge definition throughout the high-speed injury frames in consideration of brightness, contrast, and presence of blur [33, 34]. Points with visual occlusions were excluded from analyses. Since the high velocity motion lasts for less than 20 ms, despite the obscurities, only individual landmarks, not the frames, were flagged for exclusion. All the video frames depicting rotational movement were analyzed. X and Y coordinates of the five landmarks from each frame within the injury video were identified manually and exported. To account for noise in the recordings and occasional obscuring of anatomical landmarks in the videos, data from the three head landmarks were averaged, and data from the two rotational device landmarks were averaged.

#### Automated image analysis and head tracking

In this study DeepLabCut (DLC; version 2.3.9) was used for our movement-tracking framework. DLC is an open-source deep-learning based toolbox that allows markerless pose estimation, assembly, and tracking via a neural network [35, 36]. The model of head movement was trained based on ResNet-50 by labeling 20 random frames selected from 958 total frames to sample a variety of movement dynamics from the video. While frames per second, frame size, number of frames, and laboratory conditions (including lighting) remained constant throughout the videos, visibility of selected points of interest and pig skin pigmentation varied. Since the videos resulted in inconsistent visibility conditions, a separate model was created for each video. This generates models with higher in-sample accuracy but not necessarily models that should be generalized or applied to separate injury videos. To generate each model, the five anatomical landmarks used in the manual analysis were identified in all the selected training frames (Fig. 2C–D). The model was trained for 100,000 iterations with 95% training dataset and 5% test dataset of the 20 labeled frames. After training, DLC’s network evaluation indicated that the network reported test and train errors in pixels for each video model (Supp. Table 1). Importantly, these data represent the within-video tracking performance rather than generalizations across other animal videos. Each pixel equates to approximately 0.62mm under our experimental conditions based on measurement of the rotational device’s height. The models created in this study reached an average root mean squared error (RMSE) of 1.79 pixels – approximately 1.11mm – across the training datasets and 2.80 pixels – approximately 1.74mm – across the test datasets, which indicates excellent training.

The plotted data of predicted points from DLC were obtained in csv format for further analysis of the change in position and angle over time. The predictions were overlaid onto the original video for further visual validation for accuracy. DLC provided a likelihood value of how confident the software is in its prediction of each point. Points that had less than 85% likelihood in its tracking were excluded. In one video where one anatomical head landmark was obscured for approximately half of the injury recording frame, the entire anatomical landmark’s curve was excluded. To account for noise in the recordings and occasional obscuring of anatomical landmarks in the videos, data from the three head landmarks were averaged, and data from the two rotational device landmarks were averaged.

### Calculating rotational motion from manual and automated image analysis

Once coordinates for manual and automated data points were extracted for every anatomical landmark across all injury videos, centers of rotation were calculated. Center of rotation (CoR) was utilized in calculation of the angular velocities to minimize the propagation of error from one point to other points in neighboring frames. Three CoRs were calculated, using two anatomical points on the device (CoR_device_), three anatomical points on the head (CoR_head_), or all five points anatomical points (CoR_combined_). To determine the coordinates of the CoR for DLC predictions, the Euclidean distance was calculated between the CoR and the anatomical landmarks on the average of the first thirty and the average of the last thirty frames, where no movement occurs, as shown in Eq. (1). To determine the coordinates of the CoR for manual selections, the Euclidean distance was calculated between the CoR and the anatomical landmarks on the first and last frames, where no movement occurs.


1 $$r_{n}^{i}=\sqrt{{\left(x_{CoR}-x_{n}^{i}\right)}^{2}+{\left(y_{CoR}-y_{n}^{i}\right)}^{2}}$$

The CoR coordinates were adjusted to minimize the sum of differences in the Euclidean distance between the start and end frames for the appropriate landmarks using Microsoft Excel Solver Add-in (Version 16.72), as shown in Eq. (2).


2 $$\underset{x_{CoR},y_{CoR}}{\text{argmin}}\sum_{i=1}^{5}\left(r_{n}^{i}-r_{1}^{i}\right)$$

Comparisons between CoR_device_ and CoR_combined_ and between CoR_head_ and CoR_combined_ were completed (Supp. Table 2). Thereafter, CoR_device_ was utilized for all injury device calculations and CoR_head_ was utilized for all head calculations. Once the CoR was set with coordinates $x_{CoR}$ and $y_{CoR}$, the distances between each anatomical landmark with first and last frames and the CoR were calculated as in Eq. (1) and the average of those Euclidean distances resulted in a unique fixed radius, $r^{i}$, for each anatomical landmark. This process was repeated for all injury videos.

The Euclidean distance of each anatomical point between frame n and frame n+1 was calculated in Eq. (3).


3 $$d_{n}^{i}=\sqrt{{\left(x_{n+1}-x_{n}\right)}^{2}+{\left(y_{n+1}-y_{n}\right)}^{2}}$$

The angle traveled between two sequential frames was calculated from the vertex angle at the center of rotation using the following relation derived in Eq. (4).

4 $$\Delta \theta_{n}^{i}=2*{\text{sin}}^{-1}\left(\frac{0.5*d_{n}^{i}}{r^{i}}\right)$$

where $d_{n}^{i}$ was the Euclidean distance traveled between consecutive frames, $x_{n}$ and $y_{n}$ were coordinates of an anatomical landmark in frame n, $x_{n+1}$ and $y_{n+1}$ were coordinates of the same anatomical landmark in the following frame n+1, $\Delta \theta_{n}^{i}$ was the angular displacement between consecutive frames, in radians, and $r^{i}$ was the calculated radius.

Each frame in the injury videos had a duration of 2 ms so angular velocity was obtained from dividing angle traveled by the change in time (dt=0.002s) using Eq. (5).

5 $$\omega_{n}^{i}=\frac{\Delta \theta_{n}^{i}}{dt}$$

Where ω was the angular velocity in rad/s. Angular velocity was calculated for all anatomical landmarks across all injury videos. Angular acceleration was calculated as the derivative of the angular velocity curve using Eq. (6).


6 $$\alpha_{n}^{i}=\frac{\Delta \omega_{n}^{i}}{dt}$$

#### Kinematic Analyses

The time points corresponding to peak velocities were identified and used to temporally align the data. Subsequently, all five angular velocity curves were superimposed with the peak velocity standardized as time zero (Table 1). The start time for the total injury duration was obtained from each acceleration curve using zero-crossing for The end time for the total injury duration was obtained from each acceleration curve using zero-crossing for The acceleration times were determined using the negative-to-positive zero-crossing and the positive-to-negative zero-crossing from the acceleration curve. The deceleration times were determined using the positive-to-negative zero-crossing to the negative-to-positive zero-crossing from the acceleration curve. All data were statistically assessed with the one-way ANOVAs in GraphPad Prism version 10.4.1 (532). Hedge’s g effects sizes were calculated for comparisons and reported when g > 1.

Importantly, simplification of velocity curves to just their maximum value oversimplifies the data collected over time. Moreover, our small sample size may increase the likelihood of type II errors during statistical tests or the failure to detect significant difference when they do exist. To address this, we calculated mean absolute error (MAE) and CORrelation and Analysis (CORA) to measure the average difference between two velocity curves across all injury frame timepoints. MAE quantifies how well the overall shape of two curves match across all injury timepoints with larger MAE values indicating a larger difference between velocity curves. Three MAE comparisons were made: (1) DLC curve of the rotational device compared to transducer curve of the rotational device, (2) DLC curve of the head compared to DLC curve of the rotational device, and (3) DLC curve of the head compared to transducer curve of the rotational device (Table 2).

CORA, an objective evaluation method commonly used in biomechanical studies, was used to quantify how well two curves match based on corridor, phase, magnitude, and shape [37–39] as shown in (7). The corridor method $S_{\text{corr}}$ measures the deviation of the comparison curve from the inner and outer bounds of the reference curve as guided by ISO/TS 18571 guidelines [40]. The phase, magnitude, and slope scores were calculated using the methods of Enhanced Error Assessment of Time Histories (EEARTH) [41]. The phase method $S_{ps}$ measures maximal cross-correlation between two curves after time-shifting the comparison curve onto the reference curve. The magnitude/size method $S_{s}$ measures the magnitude error between the peaks of the two curves. The progression/shape method $S_{p}$ measures the average slope trend between two curves over time.


7 $$S_{\text{CORA}}=w_{\text{corr}}\left(S_{\text{corr}}\right)+w_{ps}\left(S_{ps}\right)+w_{s}\left(S_{s}\right)+w_{p}\left(S_{p}\right)$$

Three CORA comparisons were made: (1) DLC curve of the rotational device compared to transducer curve of the rotational device, (2) DLC curve of the head compared to DLC curve of the rotational device, and (3) DLC curve of the head compared to transducer curve of the rotational device (Table 3).

**Figure 3.**
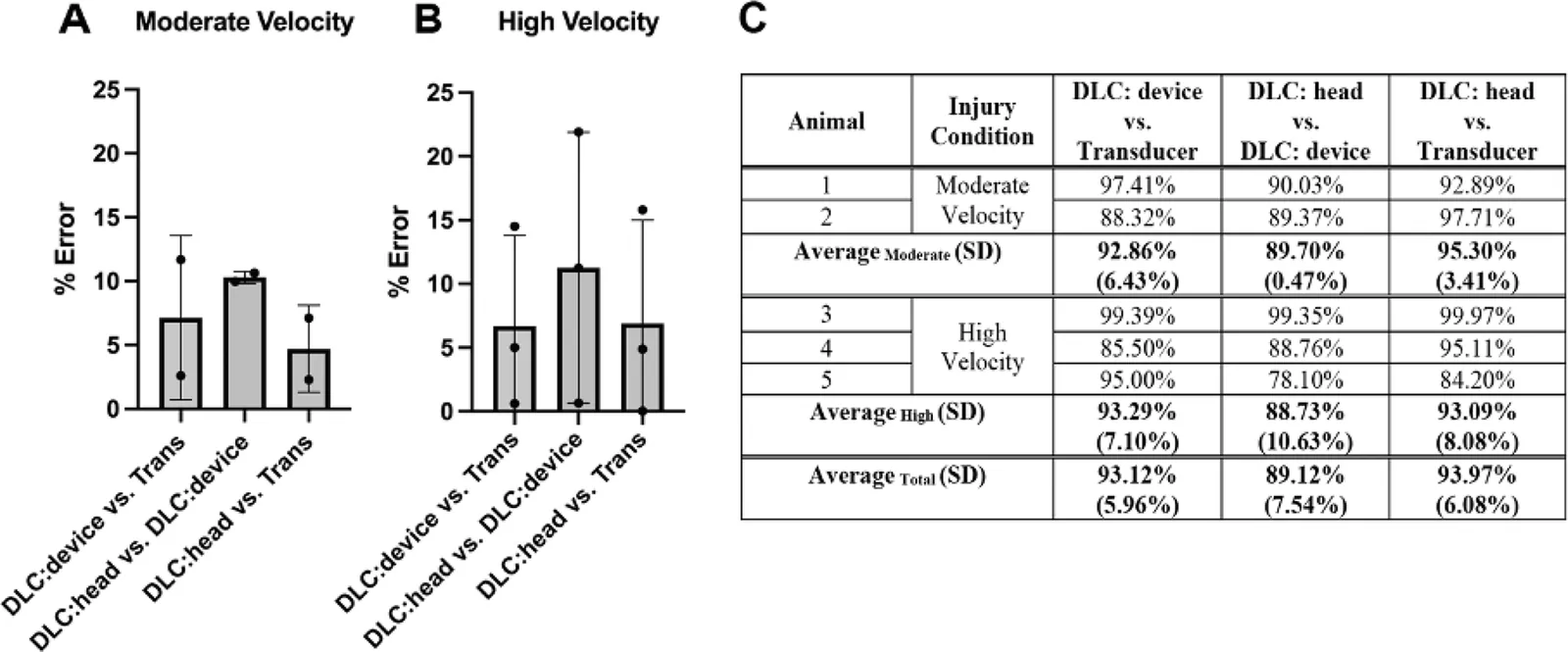
Percent error of peak angular velocities in moderate (A) and high (B) velocity injuries and percent matching of peak angular velocities (C) from the three comparisons: DLC recording of rotational device and transducer recording of the rotational device, DLC recordings of head and rotational device, and DLC recording of head and transducer recording of the rotational device.

Finally, percent matching was calculated based on peak velocity values (Fig. 3). The absolute difference in peak velocities divided by the reference peak velocity was completed for all animals. The reference peak velocity was the transducer-derived peak for all comparisons, except for the comparison between DLC: head and DLC:device. In this case, DLC: device was the reference velocity.

#### Translating porcine rotational kinematics to human concussion metrics

Peak angular velocity and minimum angular acceleration measured by the transducer were converted to human-equivalent kinematic values using conventional mass-scaling approaches [42, 43].

8 $$\omega_{\text{human}}=\omega_{\text{pig}}{\left(\frac{m_{\text{pig}}}{m_{\text{human}}}\right)}^{1/3}$$

Where $\omega_{\text{pig}}$ was the peak angular velocity of the injury the pig experienced from the transducer in radians per second, $\omega_{\text{human}}$ was the peak angular velocity in radians per second that a human would need to experience to receive the same level of injury, and $m_{\text{pig}}$ and $m_{\text{human}}$ were brain masses of pig and human brains, respectively as shown in (8).

9 $$\alpha_{\text{human}}=\alpha_{\text{pig}}{\left(\frac{m_{\text{pig}}}{m_{\text{human}}}\right)}^{2/3}$$

Where $\alpha_{\text{pig}}$ was the minimum angular acceleration of the injury the pig experienced from the transducer in radians per second (because the deceleration phase is more extreme in our model), $\alpha_{\text{human}}$ was the peak angular acceleration in radians per second that a human would need to experience to receive the same level of injury, and $m_{\text{pig}}$ and $m_{\text{human}}$ were brain masses of pig and human, respectively as shown in (9).

The female porcine rotational kinematics from each animal were scaled to female human kinematics based on the mean female adult brain mass of 1198 g and each corresponding pig brain mass, ranging from 77–88 g [44]. Given the greater representation of male athletes in studies reporting movement and injury kinematics [45, 46], the female porcine rotational kinematics were also scaled to male human kinematics using the average male adult brain mass of 1336 g and each corresponding pig brain mass for all five subjects [44].

## Results

### Kinematic recordings across injury conditions and methodologies

The current study investigated the kinematic fidelity, defined as coupling efficiency between an animal’s head and the rotational device, using traditional transducer recordings and high frame rate videography. The peak angular velocity of the rotational device recorded from the transducers was 86.87 ± 1.44 rad/s for moderate velocity injuries (target velocity range 85–90 rad/s; n = 2) and 106.75 ± 1.26 rad/s for high velocity injuries (target velocity range 105–110 rad/s; n = 3) (Table 1). The peak angular velocity of the rotational device recorded from automated DLC tracking was 80.62 ± 4.24 rad/s for moderate velocity injuries and 99.58 ± 7.35 rad/s for high velocity injuries (Table 1). The peak angular velocity of the animal’s head recorded from automated DLC tracking was 88.91 ± 4.30 rad/s for moderate velocity injuries and 110.72 ± 12.65 rad/s for high velocity injuries (Table 1).

### The head and rotational device move about the same center of rotation in videography data

Velocity was calculated as a change in distance over change in time (Eq. 3). We determined the center of rotation (CoR), calculated the respective radius for each anatomical point, and tracked change in angular position across video frames. To demonstrate that the head moves with the device, we calculated the center of rotation for the device alone (CoR_device_), for the head alone (CoR_head_), or for both objects together (CoR_combined_). Then, we compared the velocity curves using either the CoR_device_ or the CoR_combined_ for the two rotational device points. Likewise, we compared the velocity curves using either the CoR_head_ or the CoR_combined_ for the three head points. To assess curve similarities, we completed CORA, a method to assess curve matching, and found that for moderate velocity injuries, the velocity curves exhibited excellent matching for the injury device of 0.98 or higher and for the head of 0.97 at each anatomical point (Supp. Table 2). Likewise, we found that for high velocity injuries, the velocity curves exhibited excellent matching for the injury device of 0.98 or higher and for the head 0.99 or higher at each anatomical point. These data indicate that the head and rotational device are strongly coupled during the injury, both rotating around the same CoR. Despite the fact that head and rotational device rotated around the same CoR, we proceeded all subsequent analysis with head-specific and device-specific CoR, CoR_head_ and CoR_device_ respectively, in order to minimize propagration of error generated by this assumption.

### Comparison of videography-acquired and transducer-recorded device kinematics

High frame rate videography of the rotational device was compared to transducer recordings of the rotational device to further understand the reliability of reporting kinematics across methodologies (Fig. 2C–D). High frame rate videography was quantified with two different methods – manual tracking of movement and automated DLC tracking of movement – to ensure that automated DLC tracking functioned properly (Fig. 2C–F; Supp. Figure 2). Visual inspection of the manual and automated tracking appeared consistent. Furthermore, the shape of the manual and DLC angular velocity curves matched each other (Supp. Figure 2). We calculated MAE and CORA, comparing the manual tracking of the device to the transducer recording (Supp. Table 3). In parallel, we calculated MAE and CORA, comparing the DLC tracking of the device to the transducer recording (Table 2; Table 3).

Both tracking methods demonstrated comparable performance across conditions. In moderate velocities, manual and DLC tracking achieved MAEs of 5.97 rad/s and 7.19 rad/s, and CORA scores of 0.94 and 0.91, respectively. Similarly, for high velocities, manual tracking produced an MAE of 15.49 rad/s and a CORA of 0.78, compared to 12.76 rad/s and 0.84 for DLC tracking (Table 2; Supp. Table 3). These data suggested that automated tracking of video data was feasible with DLC and generated a similar level of accuracy as manual tracking throughout injury frames.

**Figure 4.**
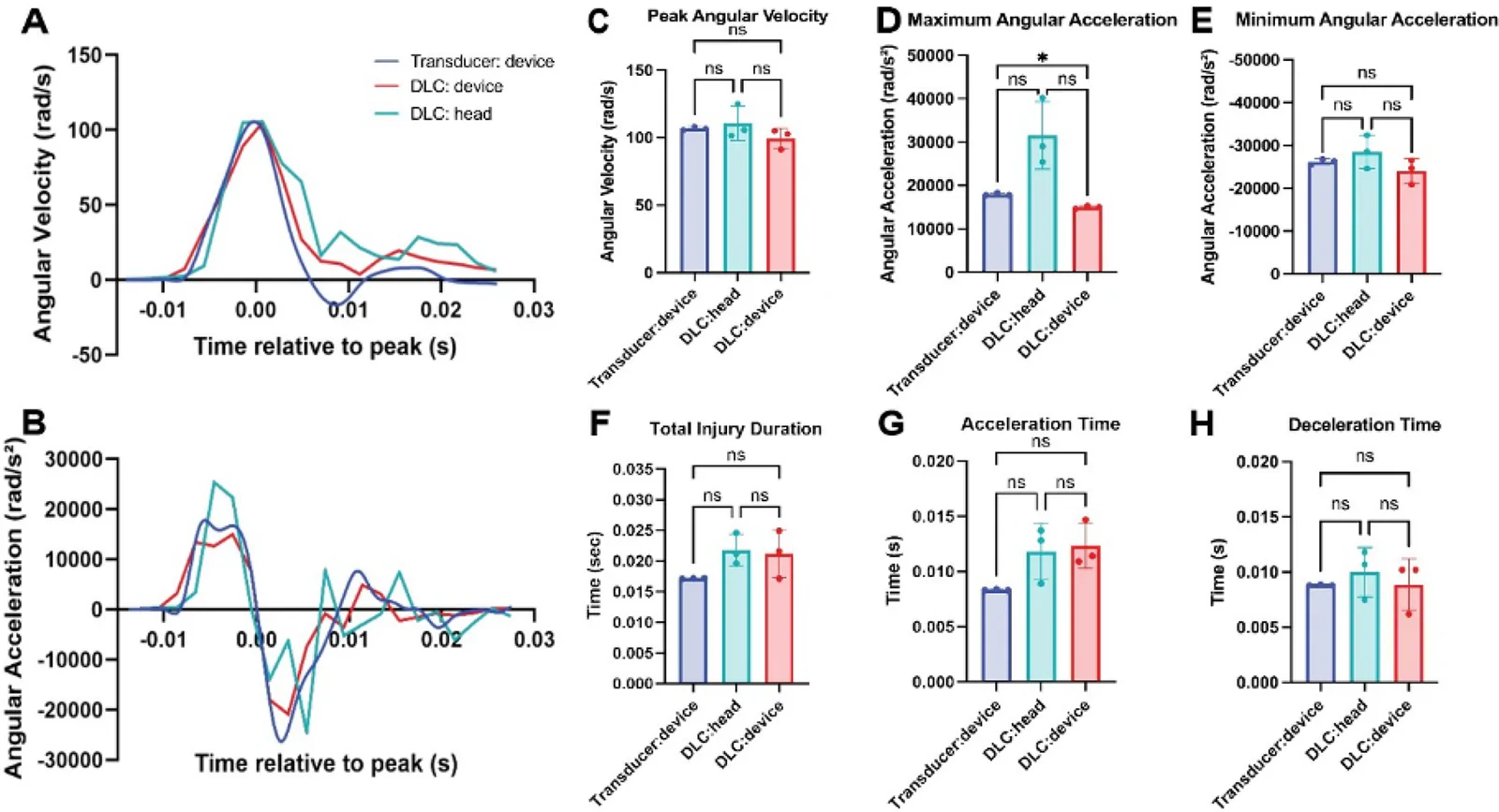
Example angular velocity and acceleration curves of the rotational device collected from transducer recordings and DLC recordings (A-B). Comparisons of peak angular velocity (C), maximum angular acceleration (D), minimum angular acceleration (E), total injury duration (F), acceleration time (G), and deceleration time (H) between transducer recording and DLC recording of the rotational device and head in high velocity injuries. Waveforms have been peak-aligned for comparison. Data represent mean ± standard deviation.

**Figure 5.**
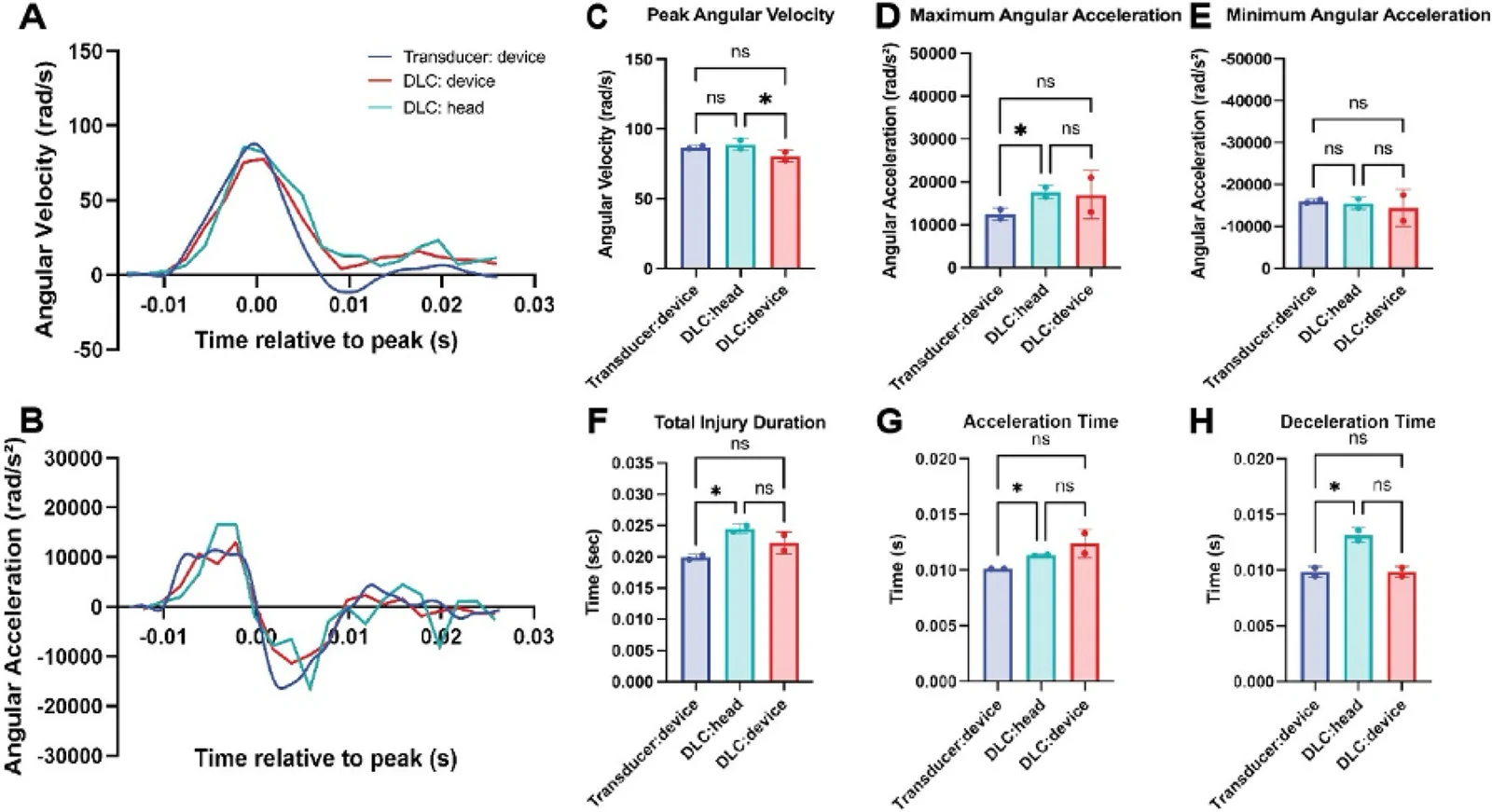
Example angular velocity and acceleration curves of the rotational device collected from transducer recordings and DLC recordings (A-B). Comparisons of peak angular velocity (C), maximum angular acceleration (D), minimum angular acceleration (E), total injury duration (F), acceleration time (G), and deceleration time (H) between transducer recording and DLC recording of the rotational device and head in moderate velocity injuries. Waveforms have been peak-aligned for comparison. Data represent mean ± standard deviation.

To further determine the validity of automated tracking of movement with DLC, we compared DLC-extracted automated data to transducer data. The rotational device’s angular velocity curves demonstrated strong correlations whether recordings were generated from transducer or DLC data (Fig. 4A; Fig. 5A; Supp. Figure 3). Likewise, angular acceleration of the rotational device generated similar curves across methods, although slightly less coupled than velocity curves (Fig. 4B; Fig. 5B).

The two methods showed strong kinematic fidelity with no significant difference in peak angular velocity (moderate velocity: g=−1.13; Fig. 4C; Fig. 5C). We did detect a significant difference in maximum angular acceleration in high velocity injuries (p = 0.026; g=−7.33; Fig. 4D) but not moderate velocity injuries (g = 0.62; Fig. 5D). Like the angular velocity trends, the minimum angular acceleration, total injury duration, acceleration time, and deceleration time between the two methods showed no signficiant differences in moderate or high velocity injuries (high velocity: g = 1.17 for total injury duration, g = 2.19 for acceleration time; moderate velocity: g = 1.01 for total injury duration, g = 1.46 for acceleration time; Fig. 4E–H; Fig. 5E–H). Given that our data set may be prone to generating type II statistical errors, we also assessed holistic waveform validation using MAE–a metric of average error across the whole curve–as well as CORA–a method to assess curve matching. Quantifying the coupling of rotational device velocity curves between the transducer and DLC data yielded MAEs of 7.19 ± 3.31 rad/s for moderate velocity injuries and 12.76 ± 3.23 rad/s for high velocity injuries (Table 2).

CORA scores of the rotational device’s movement via the transducer and DLC were 0.91 for the moderate velocity injuries and 0.84 for the high velocity injuries, indicating good to excellent curve agreement (Table 3). The difference of the peak angular velocities between the automated DLC tracking of the rotational device and transducer recording of the rotational device was 7.14 ± 6.43% for moderate velocity injuries and 6.71 ± 7.10% for high velocity injuries (Fig. 3; Supp. Figure 4). MAE and CORA were also completed on acceleration curves of the transducer and DLC data (Supp. Table 4; Supp. Table 5). In general, acceleration curves had a relatively higher MAE and a lower CORA score compared to velocity data.

Taken together, the strong overall alignment of the curve profiles, low MAE scores, and high CORA scores suggest that the rotational device’s peak angular velocity was captured with consistent kinematic fidelity across videography and transducer recordings. Thus, throughout both moderate and high velocity injuries, these data suggest that automated DLC video tracking of the rotational device reliably mirrored transducer tracking of the rotational device.

### Comparison of head movement and device movement during injury

A primary goal of this study was to understand the kinematic relationship between head and rotational device during a closed-head rotational acceleration TBI in pigs. Given that high frame rate videography can capture injury dynamics and can be reliably analyzed with automated methodologies (DLC), we next considered the kinematic coupling efficiency between the head and rotational device during an injury. To assess these relationships, we utilized DLC-analyzed videography data of the head compared to DLC-analyzed videography data of the rotational device. The angular velocity curve of the rotational device closely matched the angular velocity curve of the head (Fig. 4A; Fig. 5A; Supp. Figure 3). While there were some variations, angular acceleration of the head typically followed the trend of the rotational device (Fig. 4B; Fig. 5B). Peak angular velocity was not significantly different during high velocity TBI (g=−0.86; Fig. 4C) but was significantly lower for the injury device during moderate velocity TBI (p = 0.021; g = 1.11; Fig. 5C). Other key kinematics terms including maximum angular acceleration, minimum angular acceleration, total injury duration, acceleration time, and deceleration time between the rotational device and head were not significantly different during moderate or high velocity injuries (high velocity: g = 2.42 for maximum acceleration, g = 1.04 for minimum acceleration; moderate velocity: g = 3.31 for deceleration time; Fig. 4D–H; Fig. 5D–H).

Again, overall curve differences were assessed with MAE and CORA. The coupling between the head and the rotational device resulted in MAEs of 6.26 ± 0.96 rad/s for moderate velocity injuries and 12.42 ± 1.83 rad/s for high velocity injuries (Table 2). The difference of the peak angular velocities between the automated DLC trackings of the head and the rotational device was 10.30 ± 0. 47% for moderate velocity injuries and 11.27 ± 10.63% for high velocity injuries (Fig. 3; Supp. Figure 4). Furthermore, CORA scores between the curves were 0.90 for moderate velocity injuries and 0.83 for high velocity injuries (Table 3), suggesting good to excellent curve matching. MAE and CORA scores of the acceleration curves had a relatively higher MAE and a lower CORA score compared to velocity data (Supp. Table 4; Supp. Table 5).

Peak percent error, MAE, and CORA values of velocity data exhibited a similar magnitude to previous comparisons between the transducer and automated DLC recording of the rotational device. Together, these data suggest that automated DLC tracking of the head movement mirrored the rotational device movement across these rotational parameters and exhibited variability in-line with these methodologies.

### Evaluating transducer accuracy of head kinematics during rotational TBI

DLC-tracking of videography data demonstrated a high degree of kinematic coupling between the rotational device and subject’s head. However, videography tracking may be infeasible or offer an insufficient data sampling rate in future studies. Historically, our team and others have utilized transducers mounted to the rotational device in order to infer injury speeds of the subject’s head [7, 10, 13–15, 17, 26, 47]. Thus, we sought to determine if data from transducers mounted to the rotational device alone, could approximate head movement kinematics during a TBI. Comparing transducer data of the rotational device to DLC data of the head, we found the angular velocity of the head and rotational device were closely matched over time (Fig. 4A; Fig. 5A; Supp. Figure 3). However, angular acceleration of the head demonstrated some curve separation from the rotational device–especially during the acceleration phase (Fig. 4B; Fig. 5B). In line with these observations, there was no significant difference in peak angular velocity between the transucer and the head for either moderate or high velocity injuries (high velocity: g=−1.09; Fig. 4C; Fig. 5C). However, there was notable, but not significant changes in maximum acceleration during a high velocity injury (g = 1.99; Fig. 4D) and significant changes in maximum acceleration during moderate velocity injury (p = 0.021; g = 1.96; Fig. 5D). Minimum acceleration, total injury duration, acceleration time, and deceleration time showed no significant differences between the head and transducer recording during high velocity injuries (high velocity: g = 2.05 for total injury duration, g = 1.54 for acceleration time; Fig. 4E–H). While minimum acceleration was not significantly different, total injury duration (p = 0.026; g = 4.26; Fig. 5F), acceleration time (p = 0.032; g = 14.29; Fig. 5G), and deceleration time (p = 0.024; g = 3.31; Fig. 5H) were increased for the head during moderate velocity injuries.

The MAE between the head and transducer velocity curves was 10.12 ± 4.33 rad/s for moderate velocity injuries and 20.67 ± 4.71 rad/s for high velocity injuries (Table 2). CORA scores between the head and rotational device were 0.86 for the moderate velocity injuries and 0.74 for the high velocity injuries, indicating fair to good curve agreement (Table 3). Peak angular velocity measured from automated DLC head tracking was within 4.70 ± 3.41% of transducer-recorded values for moderate velocity injuries and within 6.91 ± 8.08% for high velocity injuries (Fig. 3; Supp. Figure 4). MAE and CORA scores of the acceleration curves had a relatively higher MAE and a lower CORA score compared to velocity data (Supp. Table 4; Supp. Table 5). Overall, peak percent error, MAE, and CORA scores indicated slightly poorer matching relative to previous comparisons. However, scores still indicated a good degree of matching. Given that peak angular velocity and minimum angular acceleration metrics were largely conserved, changes in the MAE and CORA matching were likely influenced by mismatch during the positive acceleration phase.

Together, the overall alignment of the curve profiles, peak percent error, moderate MAE scores, and high CORA scores demonstrate a high degree of kinematic fidelity between the rotational device-mounted transducer and the head kinematics during sagittal rotations at these injury speeds, especially for peak angular velocity.

### Scaled animal rotational kinematics fit within existing human concussion data

**Figure 6.**
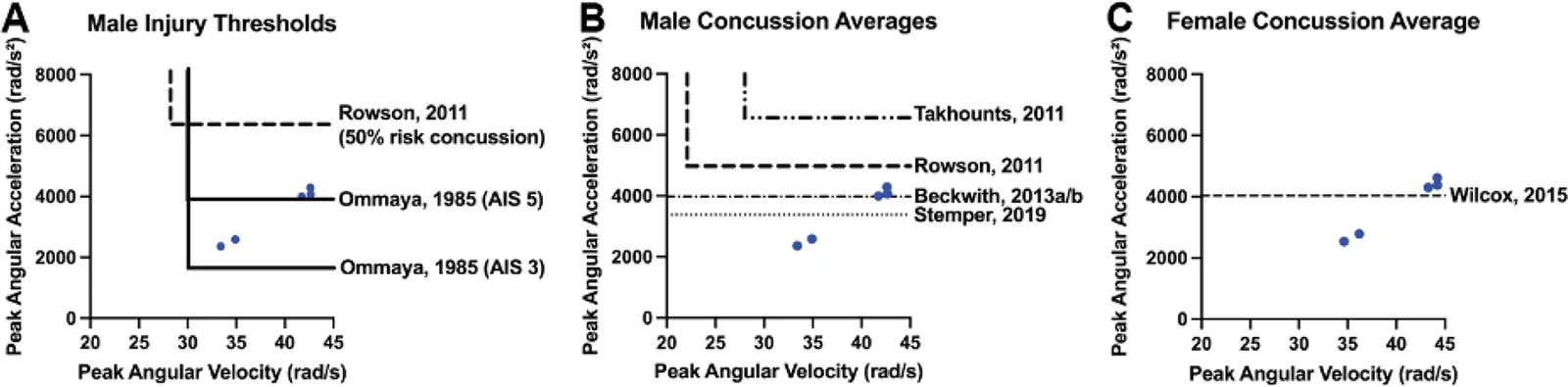
Scaled angular kinematics data of five animal subjects (denoted as blue dots) compared to male injury thresholds (A), male concussion averages (B), and female concussion average (C). Lines represent distinct thresholds denoted by each listed study or author. Only studies with concussion diagnosis and angular kinematics were included.

To contextualize the injury severity that animals experienced, rotational parameters of the rotational device were scaled to approximate the kinematics that would have been experienced by a larger, human brain. Scaling injury kinematics to human equivalents enables interpretation of injury severity by allowing comparisons to established injury thresholds and typical biomechanical profiles observed in clinical populations. Researchers have utilized mass scaling equations to correlate brain injury loads between humans and animals [8, 42, 43]. For each animal, peak angular velocity and minimum angular acceleration were calculated with respect to adult male and adult female average brain masses (Table 4). Minimum angular acceleration was utilized because it exhibited stronger kinematic matching between the head and transducer and because minimum acceleration typically exhibits a larger magnitude in this model. Scaled peak angular velocity and minimum angular acceleration were plotted in comparison to human concussion studies (Fig. 6). Only studies with rotational kinematics (angular velocity/acceleration) and concussion diagnosis were included. Previous studies established thresholds that predicts a certain level of brain injury in humans. Severity of damage to the brain after TBI is ranked on the Abbreviated Injury Scale (AIS), ranging from 1 being minor to 6 being maximal [48]. *Ommaya* used primate inertial injury models to suggest a human clinical threshold of 1,700 rad/s^2^ for AIS 3 and of 3900 rad/s^2^ for AIS 5 injuries when rotational velocity is greater than 30 rad/s in the sagittal plane [43]. Rowson *et al*. utilized human head impact data to estimate a threshold of 6,383 rad/s^2^ associated with 28.3 rad/s for 50% risk of concussion [49]. While the subjects did not meet the threshold proposed by Rowson *et al*., two subjects that experienced moderate velocity injuries fit within lower clinical impact and three subjects that experienced high velocity injuries fit within more severe clinical impact category (Fig. 6A).

Most human studies are observational in nature, meaning they do not control the injury mechanism and severity, resulting in wide variability across studies and cohorts. Thus, most studies report the average rotational kinematic metrics from the concussion-diagnosed adults [50, 51]. Takhounts *et al*. reported average of 6,572 rad/s^2^ associated with 28 rad/s in 12 male college football players, and Rowson *et al*. reported average of 4,986 rad/s^2^ associated with 22.1 rad/s in 33 male football athletes whose impacts were primarily in the sagittal plane [49, 52]. However, Beckwith *et al*. reported an average of 3,977 rad/s^2^ in 105 athletes, and Stemper *et al*. reported an average of 3,379 rad/s^2^ from 50 concussions [53, 53, 54]. Three higher velocity injuries were above the reported averages from Stemper *et al*. and Beckwith *et al*., but the two moderate velocity injuries experienced lower peak angular acceleration compared to the reported averages (Fig. 6B).

We also wanted to draw a comparison of female animals to women. However, only one study fit our inclusion criteria. Wilcox et al. reported that four subjects were diagnosed with concussion from an identifiable single impact and the average peak angular acceleration was 4,030 ± 1,435 rad/s^2^ [55]. Three high velocity injuries exceeded the average peak angular acceleration. Animal #2 was within one standard deviation of this threshold, but Animal #1 was not (Fig. 6C). Together, these data suggested that injurious kinematics generated during the high velocity procedure, exceed scaled injury thresholds. However, injury kinematics employed at the moderate velocity procedure exceeded some injury thresholds but did not surpass male or female concussion averages.

## Discussion

Accurate recording and reporting of injury kinematics are critical to understanding the relationships between injury biomechanics and outcomes [56]. Here, this study aims to determine the recording accuracy of injury kinematics in an established large animal model of closed-head diffuse TBI. Utilizing hydro-magnetic transducers and high frame rate videography, we were able to compare rotational device kinematics to head kinematics. We report that the head has a very high kinematic coupling, i.e. 93 to 95% matching of peak angular velocity on average, to the rotational device when subjects are injured in the sagittal plane within these rotational velocities. The angular velocity curves across all subjects showed strong correlations between the head and rotational device motion across measurement methodologies. Kinematic fidelity such as peak angular velocity, minimum angular acceleration, and velocity curve trajectory, was conserved across lower and higher angular velocities. These findings generate confidence in historical reports utilizing similar methods to report injury kinematics across pathology, physiology, and behavior [8, 13, 14, 17, 25, 47, 57–69].

While angular velocity trends exhibited a high degree of kinematic fidelity, angular acceleration curves exhibited a slightly lower degree of kinematic coupling. While rapid change in velocity contributes to extent of tissue damage and subject’s experience, we find that acceleration values derived from velocity curves amplify noise in the velocity curves. Indeed, calculating acceleration from a limited number of velocity datapoints, as is the case for the video-acquired data, can magnify errors in the data. Although there is less degree of kinematic coupling in angular acceleration, particularly maximum acceleration, the velocity curves and minimum acceleration closely match across injury severities and timepoints. Future studies where time-synched head- and rotational device-mounted accelerometers on complete cohorts will be required to delineate the true acceleration effects of the head.

Previous studies imparting high velocity injuries to pigs in the coronal plane have suggested that kinematic measurements from the rotational device may significantly overestimate the true extent of head movement during rotational acceleration [18–20]. For example, Mayer *et al*. found 51% fidelity of peak angular velocity in the coronal plane between the rotational device and the animal subject’s head [19]. This drastic difference in kinematic fidelity between our study and Mayer *et al*. could be attributed to factors such as plane of rotation, body positioning, craniofacial load distribution, maxillofacial integrity, and higher peak angular velocities. Indeed, rotation in the coronal plane requires significantly higher peak angular velocity and acceleration to generate tissue strain and pathology relative to sagittal injuries. When applying a range of peak angular velocities to subjects during coronal rotation, the degree of coupling efficiency was inversely proportional to the peak angular velocity [20]. However, even at a similar target peak angular velocity of 110 rad/s, the coupling efficiency in the current study was much higher during rotations in the sagittal plane relative to the previous study of the coronal plane [20]. Given the data available, it remains unknown if methodological or anatomical differences account for differences in coupling efficiency across rotational planes. The maxillofacial structure may transmit forces more effectively when upward loads are applied symmetrically across the maxilla, as in sagittal rotations, compared to asymmetrical loading on one side of the maxilla, as in coronal rotations. Symmetrical load distribution could enhance kinetic fidelity and coupling between the head and the rotational device during sagittal loading. Importantly, fractures to the craniofacial or snout bones could drastically affect energy transfer efficiency from the rotational device to the animal’s head as fractured bones will not completely transmit forces from the rotational device to the head and will instead rely on cartilaginous and soft tissues to transfer forces to the calvaria. Here, we report data from a cohort of animals that had no detectable fractures post-injury, as confirmed at the point of brain extraction.

Findings from this study indicated that transducer data has been and can continue to be a valid approximation of head movement especially for maximum velocity and minimum acceleration during rapid rotational injuries in the sagittal plane. The rotational device-mounted transducers have a substantially higher resolution as they record the electrical voltage changes at 10 kHz. In contrast, videography data collection using our current equipment is much slower, collecting video frames at 500 Hz. The capabilities allow the transducer to measure with higher temporal resolution, capturing rapid changes in motion more precisely than video-based tracking methods. Furthermore, mathematical derivation of already fewer data points from videography data could oversimplify rotational movement, propagating error even from a minor deviation in a single coordinate from its intended position. Thus, videography tracking may be infeasible and/or offer an insufficient data sampling rate in future studies. Nevertheless, the close alignment in velocity profiles over time regardless of methodology supports the utility of video tracking and transducer recording for general motion analysis, especially for experiments that are not conducive to sensor mounting.

In previous studies, a binomial smoothing function was used to reduce noise in the transducer signal [10, 13, 14, 63]. While this method reduced some noise, it was unable to adequately isolate the signal. In the current study, we updated the filtering methodology to a more robust and standard technique by implementing a high-attenuation filter. Specifically, a fourth order low pass Butterworth filter with a cutoff frequency of 300 Hz was applied for transducer data post-processing. This filter method is comparable to the CFC filters, specifically the CFC 180 filter, recommended by SAE J211 standards’, which details filtering standards used in vehicle manufacturer crash testing [28, 70]. As the rotational motion induced in this study modeled a whiplash-like injury commonly seen in motor vehicle crashes, the use of data filtering comparable to SAE J211 standards allows for more direct comparison with automotive crash test data. In addition, previous literature detailed that the Butterworth filter equivalent of the CFC 180 filter with a cutoff frequency of 300 Hz was optimal for filtering noise from angular velocity measurements taken outside of the head’s center of gravity as done in this study [28].

We utilized MAE to quantify the average deviation of velocity and acceleration curves across the entire injury. Comparing peak kinematic terms across conditions allowed us to determine potential differences in the peak of the curve. In contrast, MAE allowed us to quantify the extent of matching across two curves throughout the injury recording. In general, MAE was relatively low in reference to the dataset, indicating a good degree of matching between methodologies and between the head and rotational device. However, for most comparisons, the difference in peak angular velocity between curves was smaller than the reported MAE between curves. This suggest that differences that result in a slightly higher MAE values come from the beginning and/or end of the velocity curves, not the middle, peak values. This is important since the peak angular velocity is a critical kinematic term used to describe and contextualize injury severity. Also, we utilized CORA to quantify how well two curves matched each other. Since the curves were aligned by their peaks due to the lack of time synchronized curves, the phase shift score S_ps_ of 1 is artificial. Peak-based magnitude score was used to calculate S_s_ in accordance with the historical usage of peak angular velocity as a metric of injury severity. While default weights of S_ps_ and S_s_ were preserved to stay aligned with the standard practice, this must be taken account in evaluation of CORA scores [38]. This study reports CORA scores ranging from 0.66–0.93, which represents fair to good matching of transducer recording of the device movement and the automated recording of the head movement [39, 71, 72].

To interpret the severity of this preclinical model within the context of human head injury biomechanics, we applied a scaling relationship to the preclinical kinematic data. While multiple scaling methods exist, the conventional mass scaling approach was chosen due to its basis in physical properties and the availability of known pig brain masses for each trial [49, 73]. Researchers report different concussion averages and propose varying injury thresholds in human literature due to variability in mechanism and injury severity. Although many injury criteria involve linear kinematics, rotational kinematics are considered to be the dominant descriptors of injury in our model so only human literature that included rotational kinematics were considered. Furthermore, there is limited human literature with both concussion diagnosis and rotational kinematics data. Scaled male data were compared with kinematic thresholds developed by Ommaya and Rowson *et al*., and average concussive values reported by Takhounts *et al*., Rowson *et al*., Beckwith *et al*., and Stemper *et al*. [43, 49, 53, 54, 74]. Kinematic data was recorded by instrumented helmets in these studies; however, there are limitations in accuracy of helmet-based sensors [75–78]. While direct comparisons of preclinical injury kinematics should not be extrapolated to specific human outcomes, all preclinical trials fell into a range that was associated with human deficits. Although the scaled data did not meet 50% AIS2^+^ injury risk threshold proposed by Rowson *et al*., the human subjects involved experienced direct impact either to the head or body, whereas animals in this study experienced purely inertial rotational injury of the head [49]. Scaled female data were compared with an average concussive angular acceleration value reported by Wilcox due to lack of threshold data [55]. Upon comparison, the scaled female data were representative of concussive injuries as determined by the surpassing of the literature concussive average. Only one subject’s kinematic parameters were beyond the average proposed by Wilcox *et al*. by more than one standard deviation. Together, these trends indicate that these injury levels were generally associated with the levels humans experience, although human injury kinematics and conditions vary widely.

As mentioned above, injury parameters are primary factors in the extent of anatomical pathology. Previous reports have detailed the neuropathological outcomes seen in pigs introduced to sagittal plane TBI at these velocities. At these injury levels, our group and others have reported damaged white matter, permeabilized neuronal membranes, reactive immune cells, leaky blood brain barrier, altered hippocampal physiology, and perturbed systemic physiology [10, 14, 17, 47, 63, 79]. Subjects that experienced moderate rotational velocities in the sagittal plane, such as those used herein, exhibited no macroscopic changes, but high velocities occasionally generated subdural and periventricular microhemorrhages with blood-brain barrier disruption at subacute times post-injury [14, 62, 66].

While this study provides valuable insights into the kinematic fidelity of the HYGE rotational device used in a pig TBI model, there are several limitations that should be considered when interpreting the results. First, the camera’s frame rate of 500 fps was significantly lower than the transducer data acquisition rate and fell short of the recommended minimum of 1000 fps for velocity tracking. This lower frame rate limited the amount of videography data collected during each injury but allowed for the capture of frames with sufficient image clarity [80]. Second, the resolution of the video introduced occasional challenges in tracking anatomical landmarks through high-speed injury frames with points in rare instances obscured by motion blur and visual occlusions. Third, since the peaks in the velocity curves were manually aligned due to the frame rate and duration differences, loading delay between machine and head could not be quantified. Although peak angular velocity has been the major component in assessing injury severity, studies show that temporal duration also plays a role in injury severity [20]. Acceleration and deceleration times were calculated to approximate temporal data from our videography data. However, higher frame rate videography that reduces motion blur and improves resolution would be recommended for future studies to acquire videography data that allows time-synchronization to transducer data. Fourth, anatomical landmarks on the skin were utilized, which can be challenging to identify and can move relative to the skull. Landmarks with visibly minimal skin-to-skull movement were selected to preserve pathology and most accurately represent skull movement. However, further validation of the relationship between scalp, skull, and brain movement is necessary. Some work has been done in humans, but it is unclear how this translates in pigs [81]. Fifth, injury conditions tested within this project were constrained to sagittal rotations ranging from 85.8 to 108.1 rad/s. Given the pathology ranging from no macroscopic changes to microhemorrhages, the selected velocity range provides a variety in pathology that seem to demonstrate a wide range of injury severities without causing immediate death. Additional studies are required to determine if these trends are conserved in different rotational planes and injury speeds. Sixth, the sample size was relatively small. Given the small sample size, the statistical tests must be interpreted with caution as it may generate type II errors where statistical differences are not detected. To account for this, we incorporated CORA and MAE metrics to bolster evaluation of overall curve trajectories. Increasing the sample size to include more replicates across and within injury conditions could help determine the reproducibility and limitations of measurement metrics. Seventh, only female pigs were included in this study. Differences between male and female head anatomy likely have negligible effects on the maximum rotational velocity experienced; however, this should be determined empirically in the sagittal plane [20, 26]. Finally, a mass scaling method was used for the purpose of contextualizing pig data onto human literature. More advanced scaling methods exist for future studies intending to establish a correlation between pig and human kinematics data [67, 73, 82].

Overall, these findings indicate that transducer data mounted to the rotational device provides a reliable approximation of head kinematics during moderate and high-speed sagittal rotations. This work confirms this kinematic recording strategy, supporting both prior reports and future studies that employ this methodology in these contexts. Collectively, these results support the use of rotational device-mounted transducers as a close approximation of the rotational motion of the head movement in the sagittal plane within these injury parameters. Precise reporting of injury kinematics in preclinical models remains essential for contextualizing, interpreting, and investigating the consequences of clinical TBI.

## Conclusion

This study used high frame rate videography to assess how closely the motion of a rotational acceleration rotational device corresponds to sagittal head movement in a pig model. On average, kinematic coupling of the peak angular velocity between the head and the rotational device was 95.30% for moderate velocity injuries and 93.09% for high velocity injuries, demonstrating strong fidelity across the tested range of rotational velocities. This suggests that the recording from the rotational device approximates head motion in the sagittal plane, improving the reliability of the pig model for studying traumatic brain injury and enhancing the understanding of injury biomechanics.

## Supplementary Material

Supplementary Files

This is a list of supplementary files associated with this preprint. Click to download.

- KinematicsTables.docx
- SupplementalFiguresandTables.docx

## Figures and Tables

**Table 1:** Peak angular velocities (rad/s) of the head and rotational device by measurement method

Animal	Injury Condition	Transducer-recorded	Video-recorded: Manual		Video-recorded: Automated (DLC)	
		Injury device	Head	Injury device	Head	Injury device
1	Moderate Velocity	85.84	90.14	82.32	91.95	83.62
2		87.89	83.16	83.64	85.87	77.62
**Average**_**Moderate**_ **(SD)**		**86.87 (1.44)**	**86.65 (4.93)**	**82.98 (0.93)**	**88.91 (4.30)**	**80.62 (4.24)**
3	High Velocity	105.54	98.93	91.37	105.58	104.90
4		106.66	106.64	110.78	101.44	91.19
5		108.05	118.92	95.27	125.12	102.64
**Average**_**High**_ **(SD)**		**106.75 (1.26)**	**108.16 (10.09)**	**99.14 (10.27)**	**110.72 (12.65)**	**99.58 (7.35)**
**Average**_**Total**_ **(SD)**		**98.80 (10.95)**	**99.56 (13.99)**	**92.68 (11.46)**	**101.99 (15.07)**	**91.99 (11.80)**

**Table 2:** Mean Absolute Error (MAE; rad/s) of angular velocity curves between listed conditions.

Animal	Injury Condition	DLC: device vs. Transducer	DLC: head vs. DLC: device	DLC: head vs. Transducer
1	Moderate Velocity	9.53	6.94	13.18
2		4.85	5.58	7.06
**Average**_**Moderate**_ **(SD)**		**7.19 (3.31)**	**6.26 (0.96)**	**10.12 (4.33)**
3	High Velocity	9.25	13.17	15.34
4		15.61	10.33	22.44
5		13.41	13.76	24.24
**Average**_**High**_ **(SD)**		**12.76 (3.23)**	**12.42 (1.83)**	**20.67 (4.71)**
**Average**_**Total**_ **(SD)**		**10.53 (4.15)**	**9.96 (3.65)**	**16.45 (7.01)**

**Table 3:** CORrelation and Analysis (CORA) scores of angular velocity curves from the three comparisons: DLC recording of rotational device and transducer recording of the rotational device, DLC recordings of head and rotational device, and DLC recording of head and transducer recording of the rotational device.

Animal	Injury Condition	DLC: device vs. Transducer	DLC: head vs. DLC: device	DLC: head vs. Transducer
1	Moderate Velocity	0.89	0.89	0.78
2		0.93	0.92	0.93
**Average**_**Moderate**_ **(SD)**		**0.91 (0.03)**	**0.90 (0.02)**	**0.86 (0.11)**
3	High Velocity	0.91	0.84	0.83
4		0.76	0.86	0.73
5		0.86	0.78	0.66
**Average**_**High**_ **(SD)**		**0.84 (0.08)**	**0.83 (0.04)**	**0.74 (0.09)**
**Average**_**Total**_ **(SD)**		**0.87 (0.07)**	**0.86 (0.05)**	**0.79 (0.10)**

**Table 4:** Predicted human male and female injury kinematics, scaled from pig data using brain mass ratios

Animal	Injury Condition	Female		Male	
		Max. Angular Velocity (rad/s)	Min. Angular Acceleration (rad/s^2^)	Max. Angular Velocity (rad/s)	Min. Angular Acceleration (rad/s^2^)
1	Moderate Velocity	34.6	−2538.3	33.4	−2360.4
2		36.2	−2782.1	34.9	−2587.1
**Average**_**Moderate**_ **(SD)**		**35.4 (1.1)**	**−2660.2 (172.4)**	**34.2 (11)**	**−2473.7 (160.3)**
3	High Velocity	44.2	−4615.1	42.6	−4291.6
4		44.2	−4379.7	42.7	−4072.7
5		43.3	−4301.5	41.7	−4000.0
**Average**_**High**_ **(SD)**		**43.9 (0.5)**	**−4432.1 (163.2)**	**42.3 (0.5)**	**−4121.4 (151.8)**
**Average**_**Total**_ **(SD)**		**40.5 (4.7)**	**−3723.4 (981.2)**	**39.1 (4.5)**	**−3462.3 (912.4)**

## References

[1] Dewan MC, Rattani A, Gupta S, Baticulon RE, Hung Y-C, Punchak M, Agrawal A, Adeleye AO, Shrime MG, Rubiano AM, Rosenfeld JV (2019) Park. Estimating the global incidence of traumatic brain injury. J Neurosurg 130:1080–1097. 10.3171/2017.10.JNS17352 29701556

[2] Hajiaghamemar M, Seidi M, Margulies SS (2020) Head Rotational Kinematics, Tissue Deformations, and Their Relationships to the Acute Traumatic Axonal Injury. J Biomech Eng 142:0310061–03100613. 10.1115/1.4046393 32073595 PMC7104750

[3] Johnson VE, Meaney DF, Cullen DK, Smith DH (2015) Animal models of traumatic brain injury. Handb Clin Neurol 127:115–128. 10.1016/B978-0-444-52892-6.00008-8 25702213 PMC4946951

[4] Meaney DF, Smith DH, Shreiber DI, Bain AC, Miller RT, Ross DT, Gennarelli TA (1995) Biomechanical analysis of experimental diffuse axonal injury. J Neurotrauma 12:689–694. 10.1089/neu.1995.12.689 8683620

[5] Meaney DF, Morrison B, Dale Bass C (2014) The Mechanics of Traumatic Brain Injury: A Review of What We Know and What We Need to Know for Reducing Its Societal Burden. J Biomech Eng 136:0210081–02100814. 10.1115/1.4026364 PMC4023660 24384610

[6] LaPlaca MC, Simon CM, Prado GR, Cullen DK (2007) CNS injury biomechanics and experimental models. Prog Brain Res 161:13–26. 10.1016/S0079-6123(06)61002-9 17618967

[7] Meaney DF, Smith DH Biomechanics of concussion. Clin Sports Med 30:19–31, vii, 2011. 10.1016/j.csm.2010.08.009 21074079 PMC3979340

[8] Atlan LS, Smith C, Margulies SS (2018) Improved prediction of direction-dependent, acute axonal injury in piglets. J Neurosci Res 96:536–544. 10.1002/jnr.24108 28833411 PMC5803402

[9] Browne KD, Chen X-H, Meaney DF (2011) Smith. Mild Traumatic Brain Injury and Diffuse Axonal Injury in Swine. J Neurotrauma 28:1747–1755. 10.1089/neu.2011.1913 21740133 PMC3172883

[10] Cullen DK, Harris JP, Browne KD, Wolf JA, Duda JE, Meaney DF, Margulies SS (2016) Smith. A Porcine Model of Traumatic Brain Injury via Head Rotational Acceleration. Methods Mol Biol Clifton NJ 1462:289–324. 10.1007/978-1-4939-3816-2_17 PMC5553045 27604725

[11] Ross DT, Meaney DF, Sabol MK, Smith DH, Gennarelli TA (1994) Distribution of forebrain diffuse axonal injury following inertial closed head injury in miniature swine. Exp Neurol 126:291–299. 10.1006/exnr.1994.1067 7925827

[12] Smith DH, Nonaka M, Miller R, Leoni M, Chen XH, Alsop D (2000) Meaney. Immediate coma following inertial brain injury dependent on axonal damage in the brainstem. J Neurosurg 93:315–322. 10.3171/jns.2000.93.2.0315 10930019

[13] Wofford KL, Grovola MR, Adewole DO, Browne KD, Putt ME, O’Donnell JC (2021) Cullen. Relationships between injury kinematics, neurological recovery, and pathology following concussion. Brain Commun 3:fcab268. 10.1093/braincomms/fcab268 34934944 PMC8684470

[14] Wofford KL, Browne KD, Loane DJ, Meaney DF (2024) Cullen. Peripheral immune cell dysregulation following diffuse traumatic brain injury in pigs. J Neuroinflammation 21:324. 10.1186/s12974-024-03317-y 39696519 PMC11657926

[15] Wolf JA, Johnson BN, Johnson VE, Putt ME, Browne KD, Mietus CJ, Brown DP, Wofford KL, Smith DH, Grady MS, Cohen AS (2017) Cullen. Concussion Induces Hippocampal Circuitry Disruption in Swine. J Neurotrauma 34:2303–2314. 10.1089/neu.2016.4848 28298170 PMC5510797

[16] Duhaime A-C (2006) Large animal models of traumatic injury to the immature brain. Dev Neurosci 28:380–387. 10.1159/000094164 16943661

[17] Harris JP, Mietus CJ, Browne KD, Wofford KL, Keating CE, Brown DP, Johnson BN, Wolf JA, Smith DH, Cohen AS, Duda JE (2023) Cullen. Neuronal somatic plasmalemmal permeability and dendritic beading caused by head rotational traumatic brain injury in pigs-An exploratory study. Front Cell Neurosci 17:1055455. 10.3389/fncel.2023.1055455 37519631 PMC10381956

[18] Mayer AR, Ling JM, Patton DA, Stephenson DD, Dodd AB, Dodd RJ, Rannou-Latella JG, Smith DH, Johnson VE, Cullen DK, Meier TB (2022) Kinsler. Non-Linear Device Head Coupling and Temporal Delays in Large Animal Acceleration Models of Traumatic Brain Injury. Ann Biomed Eng 50:728–739. 10.1007/s10439-022-02953-w 35366746 PMC9079018

[19] Mayer AR, Ling JM, Dodd AB, Rannou-Latella JG, Stephenson DD, Dodd RJ, Mehos CJ, Patton DA, Cullen DK, Johnson VE, Pabbathi Reddy S, Robertson-Benta CR, Gigliotti AP, Meier TB, Vermillion MS, Smith DH (2021) Kinsler. Reproducibility and Characterization of Head Kinematics During a Large Animal Acceleration Model of Traumatic Brain Injury. Front Neurol 12:658461. 10.3389/fneur.2021.658461 34177763 PMC8219951

[20] Patton DA, Grunig CS, McQuaid JR, Dodd AB, Pacheco MK, Ling JM, Wick TV, Sasi Kumar D, Zotev V, Kinsler RE, Arbogast KB (2025) Mayer. Relationship Between Rotational Device Kinematics and Head Kinematics in a Large Animal Model of Traumatic Brain Injury. Ann Biomed Eng. 10.1007/s10439-025-03736-9 40314897

[21] Costea R, Ene I, Pavel R (2023) Pig Sedation and Anesthesia for Medical Research. Animals 13:3807. 10.3390/ani13243807 38136844 PMC10741165

[22] Cooper PR, Moody S (1978) Neurodiagnostic studies and the management of head injury. Comput Tomogr 2:197–206. 10.1016/0363-8235(78)90043-1 309380

[23] Gennarelli TA, Thibault LE, Adams JH, Graham DI, Thompson CJ (1982) Marcincin. Diffuse axonal injury and traumatic coma in the primate. Ann Neurol 12:564–574. 10.1002/ana.410120611 7159060

[24] Micro-Nikkor, AF 60mm f/2.8 D - Review / Test Report - Analysisat <https://photozone.de/Reviews/219-micro-nikkor-af-60mm-f28-d-review--test-report?start=1

[25] Huber CM, Patton DA, Wofford KL, Margulies SS, Cullen DK (2021) Arbogast. Laboratory Assessment of a Headband-Mounted Sensor for Measurement of Head Impact Rotational Kinematics. J Biomech Eng 143:024502. 10.1115/1.4048574 32975553 PMC10782863

[26] Song H, Tomasevich A, Paolini A, Browne KD, Wofford KL, Kelley B, Kantemneni E, Kennedy J, Qiu Y, Schneider ALC, Dolle J-P, Cullen DK (2024) Smith. Sex differences in the extent of acute axonal pathologies after experimental concussion. Acta Neuropathol (Berl) 147:79. 10.1007/s00401-024-02735-9 38705966 PMC11070329

[27] Evans LJ, O’Brien WT, Spitz G, Mutimer S, Xie B, Giesler LP, Major BP, Hickey JW, Roberts SSH, Mitra B, O’Brien TJ, Shultz SR (2025) McDonald. Associations Between Instrumented Mouthguard-Measured Head Acceleration Events and Post-Match Biomarkers of Astroglial and Axonal Injury in Male Amateur Australian Football Players. Sports Med 55:1037–1049. 10.1007/s40279-024-02138-6 39562417 PMC12011967

[28] Gellner R, Begonia M, Gagliardi S, Tierney G, Rowson S (2023) Optimal Cutoff Frequencies for Filtering Linear Acceleration and Angular Velocity Signals Associated with Laboratory Head Impacts Measured with Externally Mounted Sensors., at https://www.ircobi.org/wordpress/downloads/irc23/pdf-files/2341.pdf 10.1007/s10439-024-03466-4 PMC10995032 38403749

[29] Gellner RA, Begonia MT, Wood M, Rockwell L, Geiman T, Jung C (2024) Rowson. Instrumented Mouthguard Decoupling Affects Measured Head Kinematic Accuracy. Ann Biomed Eng 52:2854–2871. 10.1007/s10439-024-03550-9 38955890 PMC11402849

[30] Safety Test Instrumentation Standards Committee Instrumentation for Impact Test Part 1 - Electronic Instrumentation 10.4271/J211/1_202208

[31] Gellner R, Begonia M, Rowson S (2024) Choosing Optimal Cutoff Frequencies for Filtering Linear Acceleration and Angular Velocity Signals Associated with Head Impacts Measured by Instrumented Mouthguards. Ann Biomed Eng 52:1415–1424. 10.1007/s10439-024-03466-4 38403749 PMC10995032

[32] WuLC, LaksariK, KuoC, LuckJF, KleivenS (2016) C. R. ‘Dale’ Bass, and D. B. Camarillo. Bandwidth and sample rate requirements for wearable head impact sensors. J. Biomech. 49:2918–2924. 10.1016/j.jbiomech.2016.07.00427497499

[33] Arbogast KB, Caccese JB, Buckley TA, McIntosh AS, Henderson K, Stemper BD, Solomon G, Broglio SP, Funk JR (2022) Crandall. Consensus Head Acceleration Measurement Practices (CHAMP): Origins, Methods, Transparency and Disclosure. Ann Biomed Eng 50:1317–1345. 10.1007/s10439-022-03025-9 35920964 PMC9652170

[34] Bailey A, Funk J, Lessley D, Sherwood C, Crandall J, Neale W, Rose N (2020) Validation of a videogrammetry technique for analysing American football helmet kinematics. Sports Biomech 19:678–700. 10.1080/14763141.2018.1513059 30274537

[35] Mathis A, Mamidanna P, Cury KM, Abe T, Murthy VN, Mathis MW, Bethge M (2018) DeepLabCut: markerless pose estimation of user-defined body parts with deep learning. Nat Neurosci 21:1281–1289. 10.1038/s41593-018-0209-y 30127430

[36] Nath T, Mathis A, Chen AC, Patel A, Bethge M (2019) Mathis. Using DeepLabCut for 3D markerless pose estimation across species and behaviors. Nat Protoc 14:2152–2176. 10.1038/s41596-019-0176-0 31227823

[37] Albert DL (2021) Variations in User Implementation of the CORA Rating Metric. Stapp Association. 10.4271/2020-22-0001 33636001

[38] Gehre C, Gades H, Wernicke P (2009) Objective Rating of Signals Using Test and Simulation Responses. Proc. 21ST ESV Int. Tech. Conf. Enhanc. Saf. Veh. HELD JUNE 2009 Stuttg. Ger., at https://trid.trb.org/View/1100058

[39] Thunert C (2023) CORAplus Release 4.1.0 User’s Manual

[40] ISO/TS 18571 (2024) 2024 - Road vehicles – Objective rating metric for non-ambiguous signalsat <https://standards.iteh.ai/catalog/standards/iso/c42e1ac3-e5d0-433b-9516-50cd7be3ac10/iso-ts-18571-

[41] Zhan Z, Fu Y, Yang R-J (2011) Enhanced Error Assessment of Response Time Histories (EEARTH) Metric and Calibration Process. SAE Int. 10.4271/2011-01-0245

[42] Holbourn AHS, THE MECHANICS OF BRAIN, INJURIES (1945) Br Med Bull 3:147–149. 10.1093/oxfordjournals.bmb.a071895

[43] Ommaya AK (1985) Biomechanics of head injury: Experimental aspects. In: Biomechanics of Trauma, edited by Nahum A. and Melvin J. W.. Norwalk, Connecticut: Appleton-Century-Crofts, pp. 245–269

[44] Hartmann P, Ramseier A, Gudat F, Mihatsch MJ (1994) Polasek. [Normal weight of the brain in adults in relation to age, sex, body height and weight]. Pathol 15:165–170. 10.1007/s002920050040 8072950

[45] Brennan JH, Mitra B, Synnot A, McKenzie J, Willmott C, McIntosh AS, Maller JJ (2017) Rosenfeld. Accelerometers for the Assessment of Concussion in Male Athletes: A Systematic Review and Meta-Analysis. Sports Med Auckl NZ 47:469–478. 10.1007/s40279-016-0582-1 27402455

[46] Tierney G (1916) Concussion biomechanics, head acceleration exposure and brain injury criteria in sport: a review. Sports Biomech. 23:1888–2024. 10.1080/14763141.2021.2016929 34939531

[47] Wofford KL, Harris JP, Browne KD, Brown DP, Grovola MR, Mietus CJ, Wolf JA, Duda JE, Putt ME, Spiller KL (2017) Cullen. Rapid neuroinflammatory response localized to injured neurons after diffuse traumatic brain injury in swine. Exp Neurol 290:85–94. 10.1016/j.expneurol.2017.01.004 28081963 PMC5529036

[48] Loftis KL, Price J (2018) Gillich. Evolution of the Abbreviated Injury Scale: 1990–2015. Traffic Inj Prev 19:S109–S113. 10.1080/15389588.2018.1512747 30543458

[49] Rowson S, Duma SM, Beckwith JG, Chu JJ, Greenwald RM, Crisco JJ, Brolinson PG, Duhaime A-C, McAllister TW (2012) Maerlender. Rotational head kinematics in football impacts: an injury risk function for concussion. Ann Biomed Eng 40:1–13. 10.1007/s10439-011-0392-4 22012081 PMC10465647

[50] McIntosh AS, Patton DA, Fréchède B, Pierré P-A, Ferry E, Barthels T (2014) The biomechanics of concussion in unhelmeted football players in Australia: a case–control study. BMJ Open 4:e005078. 10.1136/bmjopen-2014-005078 PMC4039841 24844272

[51] Pellman EJ, Viano DC, Tucker AM, Casson IR and Waeckerle J. F.. Concussion in professional football: reconstruction of game impacts and injuries. Neurosurgery 53:799–812; discussion 812–814, 2003. 10.1093/neurosurgery/53.3.799 14519212

[52] Takhounts E, Ridella S, Rowson S, Duma SM (2011) and Hasija V.. Kinematic rotational brain injury criterion (BRIC)., at https://www.researchgate.net/publication/303205877_Kinematic_rotational_brain_injury_criterion_BRIC

[53] Beckwith JG, Greenwald RM, Chu JJ, Crisco JJ, Rowson S, Duma SM, Broglio SP, McAllister TW, Guskiewicz KM, Mihalik JP, Anderson S, Schnebel B, Brolinson PG (2013) Collins. Timing of Concussion Diagnosis is Related to Head Impact Exposure Prior to Injury. Med Sci Sports Exerc 45:747–754. 10.1249/MSS.0b013e3182793067 23135364 PMC3605273

[54] Stemper BD, Shah AS, Harezlak J, Rowson S, Mihalik JP, Duma SM, Riggen LD, Brooks A, Cameron KL, Campbell D, DiFiori JP, Giza CC, Guskiewicz KM, Jackson J, McGinty GT, Svoboda SJ, McAllister TW, Broglio SP (2019) McCrea, and CARE Consortium Investigators. Comparison of Head Impact Exposure Between Concussed Football Athletes and Matched Controls: Evidence for a Possible Second Mechanism of Sport-Related Concussion. Ann Biomed Eng 47:2057–2072. 10.1007/s10439-018-02136-6 30362082 PMC6785644

[55] Wilcox BJ, Beckwith JG, Greenwald RM, Raukar NP, Chu JJ, McAllister TW, Flashman LA, Maerlender AC, Duhaime A-C (2015) Crisco. Biomechanics of Head Impacts Associated with Diagnosed Concussion in Female Collegiate Ice Hockey Players. J Biomech 48:2201–2204. 10.1016/j.jbiomech.2015.04.005 25913243 PMC4492873

[56] Keating CE (2021) Cullen. Mechanosensation in Traumatic Brain Injury. Neurobiol Dis 148:105210. 10.1016/j.nbd.2020.105210 33259894 PMC7847277

[57] Aderibigbe O, Wood LB (2025) Margulies. Cyclosporine A Accelerates Neurorecovery Transcriptional Trajectory in a Swine Model of Diffuse Traumatic Brain Injury. Int J Mol Sci 26:3531. 10.3390/ijms26083531 40331981 PMC12026708

[58] Clevenger AC, Kilbaugh T, Margulies SS (2015) Carotid artery blood flow decreases after rapid head rotation in piglets. J Neurotrauma 32:120–126. 10.1089/neu.2014.3570 25133889 PMC4291216

[59] Coats B, Binenbaum G, Peiffer RL, Forbes BJ, Margulies SS (2010) Ocular hemorrhages in neonatal porcine eyes from single, rapid rotational events. Invest Ophthalmol Vis Sci 51:4792–4797. 10.1167/iovs.10-5211 20435592 PMC2941177

[60] Eucker SA, Smith C, Ralston J, Friess SH, Margulies SS (2011) Physiological and histopathological responses following closed rotational head injury depend on direction of head motion. Exp Neurol 227:79–88. 10.1016/j.expneurol.2010.09.015 20875409 PMC3021173

[61] Friess SH, Bruins B, Kilbaugh TJ, Smith C, Margulies SS (2015) Differing effects when using phenylephrine and norepinephrine to augment cerebral blood flow after traumatic brain injury in the immature brain. J Neurotrauma 32:237–243. 10.1089/neu.2014.3468 25072522 PMC4321769

[62] Huber CM, Thakore AD, Oeur RA (2024) Margulies. Distinct Serum Glial Fibrillary Acidic Protein and Neurofilament Light Time-Courses After Rapid Head Rotations. J Neurotrauma 41:1914–1928. 10.1089/neu.2023.0660 38698671 PMC11564843

[63] Keating CE, Browne KD, Duda JE (2020) Cullen. Neurons in Subcortical Oculomotor Regions Are Vulnerable to Plasma Membrane Damage after Repetitive Diffuse Traumatic Brain Injury in Swine. J Neurotrauma 37:1918–1932. 10.1089/neu.2019.6738 32178582 PMC7462023

[64] Mull M, Aderibigbe O, Hajiaghamemar M, Oeur RA (2022) Margulies. Multiple Head Rotations Result in Persistent Gait Alterations in Piglets. Biomedicines 10:2976. 10.3390/biomedicines10112976 36428544 PMC9687234

[65] Oeur A, Mull M, Riccobono G, Arbogast KB, Ciuffreda KJ, Joshi N, Fedonni D, Master CL (2023) Margulies. Pupillary Light Response Deficits in 4-Week-Old Piglets and Adolescent Children after Low-Velocity Head Rotations and Sports-Related Concussions. Biomedicines 11:587. 10.3390/biomedicines11020587 36831121 PMC9952885

[66] Oeur A, Torp WH, Arbogast KB, Master CL (2023) Margulies. Altered Auditory and Visual Evoked Potentials following Single and Repeated Low-Velocity Head Rotations in 4-Week-Old Swine. Biomedicines 11:1816. 10.3390/biomedicines11071816 37509456 PMC10376588

[67] Pasquesi SA (2018) Margulies. Measurement and Finite Element Model Validation of Immature Porcine Brain-Skull Displacement during Rapid Sagittal Head Rotations. Front Bioeng Biotechnol 6:16. 10.3389/fbioe.2018.00016 29515995 PMC5826385

[68] Sullivan S, Friess SH, Ralston J, Smith C, Propert KJ, Rapp PE (2013) Margulies. Behavioral deficits and axonal injury persistence after rotational head injury are direction dependent. J Neurotrauma 30:538–545. 10.1089/neu.2012.2594 23216054 PMC3636580

[69] Weeks D, Sullivan S, Kilbaugh T, Smith C, Margulies SS (2014) Influences of developmental age on the resolution of diffuse traumatic intracranial hemorrhage and axonal injury. J Neurotrauma 31:206–214. 10.1089/neu.2013.3113 23984914 PMC3901955

[70] Alem N, Perry M Design of Digital Low-pass Filters for Time-Domain Recursive Filtering of Impact Acceleration Signals. United States Army Aeromedical Research Laboratory, 1995.at <https://apps.dtic.mil/sti/tr/pdf/ADA293086.pdf

[71] Giudice JS, Park G, Kong K, Bailey A, Kent R (2019) Panzer. Development of Open-Source Dummy and Impactor Models for the Assessment of American Football Helmet Finite Element Models. Ann Biomed Eng 47:464–474. 10.1007/s10439-018-02155-3 30341737

[72] Hostetler ZS, Caffrey J, Aira J, Gayzik FS (2022) Lower Extremity Validation of a Human Body Model for High Rate Axial Loading in the Underbody Blast Environment. Stapp Car Crash J 66. 10.4271/2022-22-0004 37733823

[73] Wu T, Antona-Makoshi J, Alshareef A, Giudice JS (2020) Panzer. Investigation of Cross-Species Scaling Methods for Traumatic Brain Injury Using Finite Element Analysis. J Neurotrauma 37:410–422. 10.1089/neu.2019.6576 31382861

[74] Takhounts EG, Craig MJ, Moorhouse K, McFadden J, Hasija V (2013) Development of brain injury criteria (BrIC). Stapp Car Crash J 57:243–266. 10.4271/2013-22-0010 24435734

[75] Allison MA, Kang YS, Bolte JH, Maltese MR, Arbogast KB (2014) Validation of a helmet-based system to measure head impact biomechanics in ice hockey. Med Sci Sports Exerc 46:115–123. 10.1249/MSS.0b013e3182a32d0d 23846161

[76] Holcomb TD, Marks ME, Pritchard NS, Miller LE, Rowson S, Bullock GS, Urban JE (2024) Stitzel. On-Field Evaluation of Mouthpiece-and-Helmet-Mounted Sensor Data from Head Kinematics in Football. Ann Biomed Eng 52:2655–2665. 10.1007/s10439-024-03583-0 39058402 PMC11402845

[77] Jadischke R, Viano DC, Dau N, King AI, McCarthy J (2013) On the accuracy of the Head Impact Telemetry (HIT) System used in football helmets. J Biomech 46:2310–2315. 10.1016/j.jbiomech.2013.05.030 23891566

[78] Siegmund GP, Guskiewicz KM, Marshall SW, DeMarco AL, Bonin SJ (2016) Laboratory Validation of Two Wearable Sensor Systems for Measuring Head Impact Severity in Football Players. Ann Biomed Eng 44:1257–1274. 10.1007/s10439-015-1420-6 26268586

[79] O’Donnell JC, Browne KD, Kvint S, Makaron L, Grovola MR, Karandikar S, Kilbaugh TJ, Cullen DK, Petrov D (2023) Multimodal Neuromonitoring and Neurocritical Care in Swine to Enhance Translational Relevance in Brain Trauma Research. Biomedicines 11:1336. 10.3390/biomedicines11051336 37239007 PMC10216032

[80] Gabler L, Patton D, Begonia M, Daniel R, Rezaei A, Huber C, Siegmund G, Rooks T, Wu L (2022) Consensus Head Acceleration Measurement Practices (CHAMP): Laboratory Validation of Wearable Head Kinematic Devices. Ann Biomed Eng 50:1356–1371. 10.1007/s10439-022-03066-0 36104642 PMC9652295

[81] Badachhape AA, Okamoto RJ, Johnson CL, Bayly PV (2018) Relationships between scalp, brain, and skull motion estimated using magnetic resonance elastography. J Biomech 73:40–49. 10.1016/j.jbiomech.2018.03.028 29580689 PMC5932239

[82] Wu T, Hajiaghamemar M, Giudice JS, Alshareef A, Margulies SS (2021) Panzer. Evaluation of Tissue-Level Brain Injury Metrics Using Species-Specific Simulations. J Neurotrauma 38:1879–1888. 10.1089/neu.2020.7445 33446011 PMC8219195

